# Supplementary material for: Multifunctional Geotextiles Produced from Reclaimed Fibres and Their Role in Ecological Engineering
Source: Materials (Basel). 2022 Nov 10;15(22):7957. doi: 10.3390/ma15227957 (PMC9697280; doi:10.3390/ma15227957)
Supplement: Supplementary file 1 [file materials-15-07957-s001.zip › materials-1997577-supplementary.pdf]

Table S1. List of species and their frequency in the study sites.

Species	Frequency (%)			
	I<br>n = 5	II<br>n = 7	III<br>n = 23	IV<br>n = 12
Agrostis capillaris L.		43	48	8
Agrostis gigantea Roth				33
Alnus glutinosa (L.) Gaertn.		14		
Alopecurus pratensis L.			4	67
Anagallis arvensis L.				83
Arabidopsis thaliana (L.) Heynh.				8
Armoracia rusticana P. Gaertn., B. Mey. & Scherb.				17
Arrhenatherum elatius (L.) P. Beauv. Ex J. Presl & C. Presl	60		13	
Artemisia vulgaris L.				8
Atriplex patula L.			4	
Avena fatua L.			9	
Barbarea vulgaris R. Br.				67
Bidens frondosa L.	40			8
Brassica rapa L.				58
Calamagrostis epigejos (L.) Roth			9	
Calystegia sepium (L.) R. Br.	100			
Capsella bursa-pastoris (L.) Medik.	40		4	33
Carex nigra Reichard		14		
Carex ovalis Gooden.		29		
Carex rostrata Stokes		14		
Chenopodium album L.			13	83
Chenopodium polyspermum L.				58
Cirsium arvense (L.) Scop.	80	57	61	33
Crepis biennis L.	20			8
Dactylis glomerata L.		43		
Daucus carota L.	20			17
Echinochloa crus-galli (L.) P. Beauv.				8
Elymus repens (L.) Gould		86		
Epilobium hirsutum L.	60		17	8
Epilobium tetragonum L.			26	
Equisetum arvense L.		57	65	
Erysimum cheiranthoides L.			17	
Euphorbia helioscopia L.			43	
Fallopia convolvulus (L.) Á. Löve				58
Fallopia japonica (Houtt.) Ronse Decr.				17
Festuca arundinacea Schreb.				25
Festuca ovina L.		14		
Festuca rubra L.		29		
Galeopsis tetrahit L.			4	50
Galium aparine L.		14		
Galium mollugo L.	40			
Geranium dissectum L.				33
Glechoma hederacae L.	40			
Holcus lanatus L.		86	35	25
Hordeum vulgare L.			4	

Humulus lupulus L.		43		
Hypericum maculatum Crantz		29		
Hypericum perforatum L.				17
Impatiens parviflora DC.		29	4	58
Juncus conglomeratus L.		14	9	
Juncus tenuis Willd.			13	
Lactuca serriola L.	80		57	25
Lamium album L.			14	
Lamium amplexicaule L.			4	
Lapsana communis L.	80			8
Lathyrus pratensis L.				8
Leucanthemum vulgare Lam.				8
Lolium multiflorum Lam.	20		61	100
Lolium perenne L.		14	96	75
Lotus corniculatus L.	20	14	4	8
Luzula campestris (L.) DC.		14		
Lysimachia nummularia L.		29		
Lythrum salicaria L.	60	14		
Matricria maritima L.	40			83
Medicago lupulina L.			4	33
Melandrium album (Mill.) Garcke				25
Melilotus officinalis (L.) Pall.				8
Mentha arvensis L.	40		9	8
Myosotis arvensis (L.) Hill				33
Myosotis stricta Link ex Roem. & Schult.			4	
Oxalis stricta L.	80	14		8
Papaver rhoeas L.				8
Phalaris arundinacea L.	20			25
Phleum pratense L.			17	
Plantago intermedia Gilib.			17	
Plantago lanceolata L.				17
Plantago major L.	40			8
Poa annua L.			17	25
Poa pratensis L.		43	4	
Polygonum aviculare L.			13	75
Polygonum hydropiper L.				92
Polygonum persicaria L.			17	58
Potentilla reptans L.			22	
Quercus robur L.		43		8
Ranunculus repens L.		43	57	25
Ribes nigrum L.			4	
Rosa canina L.			4	
Rubus caesius L.		57		
Rumex acetosella L.		14	39	
Rumex crispus L.				8
Rumex obtusifolius L.	80	29	52	25
Sagina procumbens L.			4	8
Salix caprea L.			9	
Salix fragilis L.			13	
Scleranthus annuus L.				8
Scrophularia nodosa L.	40	14		
Setaria viridis (L.) P. Beauv.				33
Sinapis arvensis L.	80			33

Sisymbrium officinale (L.) Scop.				8
Solidago canadensis L.	60		13	
Sonchus asper (L.) Hill				67
Spergula arvensis L.				58
Spergularia rubra (L.) J. Presl & C. Presl			4	
Stachys palustris L.	40			17
Stellaria graminea L.				33
Stellaria media (L.) Vill.			39	83
Symphytum officinale L.	80			
Tanacetum vulgare L.		14	4	17
Taraxacum officinale L.		29	35	42
Thlaspi arvense L.	60			
Trifolium pratense L.			17	25
Trifolium repens L.			52	42
Tussilago farfara L.	40	29	83	42
Urtica dioica L.		71	9	33
Vicia cracca L.		29		17
Vicia hirsuta (L.) Gray			26	42
Vicia angustifolia L.				33
Vicia sepium L.				25
Vicia tetrasperma (L.) Schreb.		29	9	25
Viola tricolor L.				42

Explanations: I – Nieboczowy, II – Międzyrzecze, III – Lipnik, IV – Wapienica, n – number of studied plots.

Table S2. The soil characteristics of the studied plots.

Site	C org. [%]	Hummus [dry mass %]	pH	[mg/100g]			[mg/kg dry mass]	
				P_2O_5	K_2O	Mg	$N\text{-}NO_3$	$N\text{-}NH_4$
Nieboczowy	1.4	2.41	5.2	5.2	16.6	23.4	22.32	2.57
	1.9	3.28	5.5	5.6	14.2	21.9	19.79	2.56
	2	3.45	5	4.7	16.3	21.2	16.2	2.58
	1.9	3.28	5.5	5.3	17.9	24	15.95	2.57
	0.7	1.21	5.9	4.3	14.8	24.4	2.49	2.49
Międzyrzecze	0.9	1.55	5.4	5.2	13.3	14.1	3.29	3.77
	1	1.72	5.9	6.7	16.3	11.4	2.42	2.42
	0.9	1.55	5.8	5.6	12.9	14.2	2.56	2.56
	1	1.72	5.4	6.2	17.7	10.8	2.37	7.55
	1	1.72	5.9	8	16.7	11.5	2.46	9.45
	1.1	1.9	5.9	5.4	13.8	10	2.45	2.66
	1	1.72	6.2	6.7	15.8	9.1	2.4	5.35
Lipnik	0.5	0.86	7.3	2.6	8.4	7.5	2.4	4.11
	0.7	1.21	7.5	2.8	8.8	6.2	2.49	6.6
	0.7	1.21	7.4	1.3	8.9	7.2	2.37	5.95
	0.9	1.55	7.4	10.7	10.8	8.3	2.34	4.61
	0.6	1.03	7	2.5	10	7.9	3.03	5.23
	0.8	1.38	6.3	1.5	10	10.1	2.5	5.16
	0.7	1.21	5.7	1.3	9.8	12.1	2.42	4.46
	0.8	1.38	5.6	1.8	12.1	9.8	2.35	4.87
	1.5	2.59	5.5	1.3	14.2	11.7	11.78	6.19
	1.4	2.41	5.6	1.6	14.9	11	15.17	12.25
	1.4	2.41	5.5	1.7	4.4	12.3	7.02	4.68

	1	1.72	5.9	4	11.6	11.3	2.41	14.38
	0.7	1.21	6.7	1.6	12.7	10	233	4.44
	0.7	1.21	7.1	2.3	12	10.1	4.26	6.92
	1.1	1.9	7.5	2.6	13.1	6.8	2.42	4.12
	0.8	1.38	7.5	2.5	13.1	6.6	2.37	5.11
	0.7	1.21	7.5	2.2	13.4	6.1	2.4	6.64
	0.9	1.55	7.4	3.5	19.5	6.7	2.48	4.79
	0.7	1.21	7.5	2	10.1	6.5	2.46	4.65
	0.7	1.21	7.4	1.4	11.6	7.6	2.41	3.2
	0.6	1.03	6.9	2.1	12.1	10.2	2.38	3.89
	0.5	0.86	4.6	1.3	8.2	15.2	2.3	3.62
	0.7	1.21	6.6	1.3	8.8	12.5	2.48	4.89
	2	3.45	5.7	3.5	8.9	8.4	0.6	10.2
	2.1	3.62	7.1	3.3	12.9	7.9	2.3	9.6
	2.4	4.14	6	3.1	9.3	9.6	2.7	11.4
	1.9	3.28	7.3	4.3	10.5	9.8	2.3	6.6
	2.8	4.83	6.2	4.2	11	10.4	2.5	11.8
Wapienica	1.6	2.76	6.4	3.2	5.2	9.1	2.5	11.3
	2.1	3.62	6	3.5	14.7	11.6	2.8	17.3
	2	3.45	7	3.5	14.2	10.1	2.3	6.1
	2.1	3.62	6	3.2	10.2	9.8	11.5	12.9
	2	3.45	7.2	4	12.1	9	2.5	10.9
	2.5	4.31	6.6	6.4	20.4	112	2.4	4.9
	2.5	4.31	7.1	6.8	13.7	11.6	2.3	6.4

Table S3. The location of vegetation centroids in DCA and the biodiversity indices (S- species richness, H-Shannon-Wiener index, E-evennes), mean height of plants in plots where Kemafil ropes (+) and control (-) and various types of geotextile were applied (wool, synthetic, control).

Site (DCA1, DCA2)	Kemafil	Material	S	H	E	Height [cm]
	+	wool	18	1.34	0.46	105.00
	+	synthetic	19	1.75	0.59	110.00
Nieboczowy (0.25; -1.97)	+	wool	14	2.03	0.77	115.00
	+	synthetic	15	2.10	0.78	100.00
	-	control	11	1.62	0.68	61.67
	+	wool	8	0.77	0.37	85.00
	+	synthetic	12	1.12	0.45	75.00
Międzyrzecze (3.36; 0.23)	+	synthetic	16	2.30	0.83	85.00
	+	wool	10	1.38	0.60	71.67
	+	wool	13	0.74	0.29	81.67
	+	wool	7	1.19	0.61	86.67
	-	control	15	2.57	0.95	50.33
	-	synthetic	8	0.70	0.34	48.33
	-	synthetic	12	0.69	0.28	48.00
	-	wool	10	0.80	0.35	63.67
	-	synthetic	7	1.95	1.00	42.00
	-	synthetic	15	0.59	0.22	62.00
Lipnik (0.042; 0.17)	-	synthetic	11	2.40	1.00	43.33
	-	synthetic	13	1.95	0.76	43.33
	-	wool	13	1.06	0.41	51.67
	-	wool	13	0.69	0.27	59.33
	+	wool	17	1.00	0.35	77.67
	+	wool	14	0.92	0.35	78.33

	+	synthetic	13	0.95	0.37	80.00
	+	synthetic	16	1.35	0.49	88.33
	+	synthetic	10	0.97	0.42	76.67
	+	synthetic	14	0.55	0.21	72.33
	+	wool	18	1.56	0.54	69.00
	+	synthetic	9	0.35	0.16	72.33
	+	synthetic	12	0.52	0.21	68.00
	+	synthetic	9	0.54	0.25	65.00
	+	synthetic	10	0.39	0.17	83.00
	-	control	8	0.46	0.22	40.00
	-	control	21	3.04	1.00	21.67
	-	synthetic	18	2.89	1.00	31.67
	+	wool	22	1.94	0.63	105.00
	+	wool	18	1.54	0.53	100.00
	+	wool	23	1.57	0.50	106.67
	+	wool	23	1.99	0.63	93.33
	+	synthetic	30	2.64	0.78	85.00
Wapienica	+	synthetic	30	2.21	0.65	86.67
(-1.23; 0.28)	+	wool	32	1.69	0.49	95.00
	+	synthetic	20	1.06	0.36	113.33
	+	synthetic	22	1.89	0.61	100.00
	+	wool	18	1.39	0.48	110.00
	-	control	42	3.46	0.93	53.33
	-	control	15	2.22	0.82	50.00

Table S4. The result of Canonical Correspondence Analysis and permutation tests of significance of environmental variables controlling species composition of vegetation (significant p-vales are in bold).

	**Conditional**			**Margin**			**Step-Forward selection**		
	**Chi-Square**	**Pseudo-F**	**P**	**Chi-Square**	**Pseudo-F**	**P**	**AIC**	**Pseudo-F**	**Pr(>F)**
Carbon	0.5447	4.6791	0.001	0.1051	0.903	0.517			
HUMMUS	0.1781	1.53	0.066	0.1053	0.9042	0.516	270.37	4.5133	**0.005**
pH	0.4988	4.2849	**0.001**	0.3871	3.3254	**0.001**	269.4	3.6054	**0.005**
P	0.4551	3.9092	**0.001**	0.3846	3.3034	**0.001**	270.46	4.5974	**0.005**
K	0.0745	0.6404	0.828	0.0564	0.4848	0.95			
Mg	0.2444	2.0993	0.071	0.223	1.9156	0.089	267.96	2.2873	0.06
HNO3	0.016	0.137	0.997	0.0195	0.1678	0.998			
HNH4	0.119	1.0221	0.405	0.1211	1.0404	0.388			
Kemafil	0.1874	1.6098	**0.05**	0.0563	0.4835	0.97			
Synth	0.1741	1.496	0.074	0.199	1.7093	**0.047**	268.65	2.9163	**0.01**
Wool	0.2356	2.0236	**0.009**	0.2356	2.0236	**0.011**	269.01	3.2425	**0.005**
Residual	4.0743								
